# Weekend admission to hospital has a higher risk of death in the elective setting than in the emergency setting: a retrospective database study of national health service hospitals in England

**DOI:** 10.1186/1472-6963-12-87

**Published:** 2012-04-02

**Authors:** Mohammed A Mohammed, Khesh S Sidhu, Gavin Rudge, Andrew J Stevens

**Affiliations:** 1 Primary Care Clinical Sciences, University of Birmingham, Birmingham, England, University of Birmingham, Edgbaston, Birmingham B15 2TT, UK; 2 Consultant in public health medicine and Honorary Senior Clinical Lecturer, Unit of Public Health, Epidemiology and Biostatistics, University of Birmingham, Edgbaston, Birmingham B15 2TT, UK; 3 Unit of Public Health, Epidemiology and Biostatistics, University of Birmingham, Edgbaston, Birmingham B15 2TT, UK

## Abstract

**Background:**

Although acute hospitals offer a twenty-four hour seven day a week service levels of staffing are lower over the weekends and some health care processes may be less readily available over the weekend. Whilst it is thought that emergency admission to hospital on the weekend is associated with an increased risk of death, the extent to which this applies to elective admissions is less well known. We investigated the risk of death in elective and elective patients admitted over the weekend versus the weekdays.

**Methods:**

Retrospective statistical analysis of routinely collected acute hospital admissions in England, involving all patient discharges from all acute hospitals in England over a year (April 2008-March 2009), using a logistic regression model which adjusted for a range of patient case-mix variables, seasonality and admission over a weekend separately for elective and emergency (but excluding zero day stay emergency admissions discharged alive) admissions.

**Results:**

Of the 1,535,267 elective admissions, 91.7% (1,407,705) were admitted on the weekday and 8.3% (127,562) were admitted on the weekend. The mortality following weekday admission was 0.52% (7,276/1,407,705) compared with 0.77% (986/127,562) following weekend admission. Of the 3,105,249 emergency admissions, 76.3% (2,369,316) were admitted on the weekday and 23.7% (735,933) were admitted on the weekend. The mortality following emergency weekday admission was 6.53% (154,761/2,369,316) compared to 7.06% (51,922/735,933) following weekend admission. After case-mix adjustment, weekend admissions were associated with an increased risk of death, especially in the elective setting (elective Odds Ratio: 1.32, 95% Confidence Interval 1.23 to 1.41); vs emergency Odds Ratio: 1.09, 95% Confidence Interval 1.05 to 1.13).

**Conclusions:**

Weekend admission appears to be an independent risk factor for dying in hospital and this risk is more pronounced in the elective setting. Given the planned nature of elective admissions, as opposed to the unplanned nature of emergency admissions, it would seem less likely that this increased risk in the elective setting is attributable to unobserved patient risk factors. Further work to understand the relationship between weekend processes of care and mortality, especially in the elective setting, is required.

## Background

Although acute hospitals offer a twenty-four hour seven day a week service, it is known that levels (seniority and numbers) of staffing are lower over the weekends than on weekdays [1]. Consequently health care processes within hospitals, such as diagnostic testing or advice from senior colleagues, may be less readily available over the weekend [2]. This "weekend phenomenon" reflects a planned reduction in elective activity as well as the notion that weekend working is unpopular amongst staff and some staff only have weekday working contracts [3]. Typically, consultants will work one weekend in four or five in English hospitals [1].

Whilst some of the operational reasons behind the weekend phenomenon may be justifiable, there appears to be some concern that outcomes for patients admitted over the weekend may be less favourable than patients admitted during the weekday [2,4] This association between weekend admission and outcome has been termed the "weekend effect"[5]. Evidence for such concern appears to stem from a few studies which have found adverse outcomes for weekend admissions [5-13]; but these studies have, in general, focused primarily on emergency admissions, have been limited to specific well defined conditions (eg stroke, myocardial infarction, aortic aneurism, chronic obstructive pulmonary disease) and/or settings (eg internal medicine, intensive care units, teaching hospital status). Nevertheless, unlike much of the previous work, we were interested in the extent to which weekend admission was associated with an increased risk of death for elective admissions separately from emergency admissions. A key justification for our work is that the mechanism which results in a weekend admission in the elective setting is a "planned" process, which is much more amenable to change because the process is primarily designed and owned by the hospital, whilst in the emergency setting the admission is "unplanned", and the underlying process is less amenable to change because of the complex interplay with out-of-hours primary care, patient behaviors and ambulance services, making a pooled analysis less justifiable. We used admission data from all acute hospitals in the National Health Service (NHS) in England to further investigate this question.

## Method

We undertook a retrospective desktop statistical analysis based on all patient discharges during the year April 2008 to March 2009 from all acute hospitals (n = 328, comprising 221 NHS Trusts) in England. The discharges were obtained via Hospital Episode Statistics [14] (HES), a routinely collected national dataset of patients admitted for care used by the NHS in England. Admissions on a Saturday or Sunday were classed as weekend admissions and admissions during Monday to Friday were classed as weekday admissions. The HES data source does not include time of admission.

Our approach was to consider elective and emergency admissions separately. For emergency cases, as recommended by Jones, [15] we excluded admissions discharged alive with a zero day length of stay, because in England such episodes of care are likely to be an artefact of the NHS drive to reduce Accident & Emergency waiting times rather than reflecting any specific clinical or epidemiological phenomenon [15].

## Data

For each discharge episode, we obtained the patient's age, gender, date of admission, date of discharge, discharged alive/dead and the Healthcare Resource Group (HRG) version 3.5. HRGs are standard groupings of clinically similar treatments which use common levels of healthcare resource [16]. They work in a similar way to the Diagnostic Related Groups [17] used in the United States and other healthcare systems primarily to support financial reimbursement to healthcare providers. We used HRG version 3.5 developed for the NHS to determine if the patient was a complex elderly patient (14/610 HRG codes ending in "99") and if the patient had complications and/or comorbidities (119/610 HRG codes that include "with cc" in their description). These "withcc" HRG codes can sometimes include an age criterion (eg aged > 69 Each admission record in the administrative HES data includes International Classification of Disease (ICD)-10 diagnoses, (one primary diagnosis and up to 19 secondary diagnoses) and procedures coded by the Office of Population Censuses and Surveys (OPCS-4) coding system, (up to a maximum of 24 per admission) which are parsed through a HRG grouping algorithm to derive the HRG for a give hospital admission.

The following HES records were excluded - day cases, patients aged less than 16 years of age on admission, episodes of care relating to maternity care (because this is a 24 h service), mental health episodes of care other than dementia (because mental health admissions are predominantly to psychiatric health hospitals).

## Descriptive statistics

We compared the case-mix profile, mortality and length of stay of patients admitted over the weekend and weekday for elective and emergency admissions using descriptive statistics. Using run charts we explored the pattern over time (month) of mortality for admissions on weekends and weekdays to determine if there were any substantial time dependent effects (eg seasonality). These descriptive analyses were also used to inform the development of a subsequent logistic regression model.

## Statistical model

We developed two logistic regression models with death in hospital as the response variable and with a binary weekend admission variable whilst controlling for the covariates shown in Table 1. The elective and emergency models shared the same covariate set except that zero day stays were included in the elective model.

**Table 1:** Covariates in the logistic regression model.

Covariate (name in model)	Levels of covariate
Age Category (agegcat)	12 groups

Complex Elderly	1 = Yes, 0 = no

Male	1 = Yes, 0 = no

HRG with comorbidities/complications	1 = Yes, 0 = no

Admission on a weekend	1 = Yes, 0 = no

Interaction: Age Category and HRG with comorbidities/complications	12 interaction terms

Admission Quarter	1 = Apr-Jun; 2 = Jul-Sep; 3 = Oct-Dec; 4 = Jan-Mar

Zero day stay†	1 = Yes, 0 = no

Weekend Admission	1 = Yes, 0 = no

† Only applicable for the elective admissions models

Age was categorised into ten five year age bands, with an index age band of 16-40 years and an ultimate age band of 90-120 years. We also tested for interaction effects between the weekend variable and other covariates (complex elderly, with comorbidity/complication, male and age category in the model) using likelihood ratio tests with p < 0.01 as the threshold for statistically significant interactions as part of the model development process. To mitigate against spurious p-values resulting from the very large sample sizes involved in our study, we undertook model development using a 10% stratified random sample from the elective and emergency admissions.

The goodness-of-fit of the model was investigated using techniques designed to test both discrimination and calibration. The discriminative ability of the models was assessed using receiver-operating characteristics (ROC) curves. The area under the ROC curve, summarised by a *c*-statistic, is a measure of the model's ability to correctly discriminate between survivors and non-survivors. The c-statistic is the probability of assigning a greater risk of death to a randomly selected patient who died compared with a randomly selected patient who survived. A value of 0.5 suggests that the model is no better than random chance in predicting death. A value of 1.0 suggests perfect discrimination. In general, values less than 0.7 are considered to show poor discrimination, values of 0.7-0.8 can be described as reasonable, and values above 0.8 suggest good discrimination. Calibration, the accuracy of risk predictions, was tested using the deciles of risk table described by Hosmer and Lemeshow, where the overall difference between expected and observed numbers of death for each decile of risk is compared using a *χ*^2^-test with 8 degrees of freedom. As this is a null hypothesis test, p-values less than 0.05 indicate evidence of significant lack of fit.

Once the initial model, based on the 10% random samples, produced adequate goodness of fit statistics (Hosmer-Lemeshow *χ*^2^-test p > 0.05) we derived the coefficients for the covariates in the initial model from all elective and emergency admissions (excluding admissions that had a zero day stay and that were discharged alive). We reproduced the goodness of fit statistics using the complete dataset model. Modelling results from the 10% random sample are shown in the appendix. All analyses were carried using STATA version 12 [18].

## Results

### Overview

There were 5,588,988 discharge episodes in the year 01/04/2008 to 31/03/2009. After excluding 948,472 emergency admissions discharged alive with a zero stay, we had 4,640,516 discharge episodes, of which 66.92% (3,105,249/4,640,516) were emergency admissions and 33.08% (1,535,267/4,640,516) were elective admissions. Table 2 shows the profiles of weekend versus weekday admissions for elective and emergency admissions. In general terms, irrespective of emergency/elective setting, weekend admissions were more likely to be male, more likely to have an HRG indicating a complex elderly patient but had similar proportions of admissions with HRGs indicating comorbidity/complications (withcc).

**Table 2:** Profile of weekday and weekend admission [SD = standard deviation]; [iqr = inter quartile range] (%) †Denominator is number of deaths in that column

Characteristic	Elective Admissions (n = 1,535,267)		Emergency Admissions (n = 3,105,249)	
	**Weekday**	**Weekend**	**Weekday**	**Weekend**

N	1,407,705	127,562	2,369,316	735,933

Male	663,295 (47.12)	63,054 (49.43)	1,124,086 (47.44)	353,665 (48.06)

**Age (years)**				

Mean Age [SD]	57.77 [17.90]	59.33 [17.73]	62.21 [21.40]	61.93 [22.01]

Median Age [iqr]	60 [27]	62 [26]	67 [34]	67 [37]

**Admission Quarter**				

1 Apr - Jun	332,598 (23.63)	32,802 (25.71)	574,134 (24.23)	183,754 (24.97)

2 Jul - Sep	361,887 (25.71)	31,790 (24.92)	585,331 (24.70)	179,880 (24.44)

3 Oct - Dec	363,746 (25.84)	30,616 (24.00)	594,647 (25.10)	180,090 (24.47)

4 Jan - Mar	349,473 (24.83)	32,354 (25.36)	615,204 (25.97)	192,209 (26.12)

**Mortality**				

Died	7,276 (0.52)	986 (0.77)	154,761 (6.53)	51,922 (7.06)

Deaths within 24 h of admission†	88 (1.21)	10 (1.01)	10,254 (6.63)	3,966 (7.64)

**Case-mix**				

HRG indicating Complex Elderly	3,932 (0.28)	404 (0.32)	179,190 (7.56)	59,961 (8.15)

HRG with comorbidity and/or complication (withcc)	192,411 (13.67)	17,688 (13.87)	842,219 (35.55)	258,807 (35.17)

**Median length of stay (days)**				

All [iqr]	1 [3]	3 [3]	4 [8]	3 [8]

Discharged alive only [iqr]	1 [3]	3 [5]	3 [7]	3 [8]

Deceased only [iqr]	12 [20]	12 [21]	8 [17]	6 [15]

**Figure 1:**
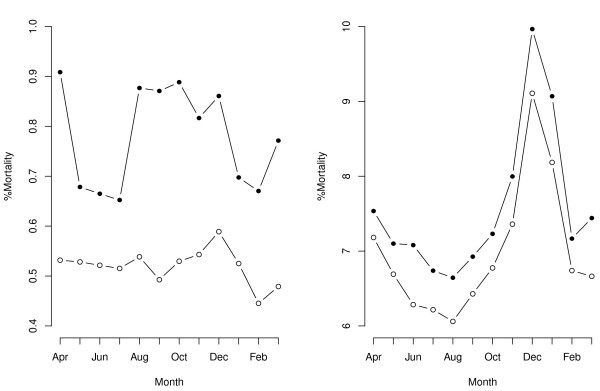
**Run charts comparing %mortality over time for elective admissions (left panel), and emergency admissions (right panel)**. The black dots are for weekend admissions and the white dots are for weekday admissions. Note the y-axes scales differ and do not include zero.

Run charts (Figure 1) comparing mortality over time for our three admission strata showed (a) that weekend admissions had consistently higher mortality over time for all admission strata, and (b) there was some evidence of seasonality with a peak in %mortality during December 2008 and a trough in August 2008, especially marked in the emergency admissions.

Below we describe the profile of elective and emergency admissions in more detail.

### Elective admissions

Of the 1,535,267 elective admissions, 91.7% (1,407,705/1,535,267) were admitted on the weekday and 8.3% (127,562/1,535,267) were admitted on the weekend. A higher proportion of weekend admissions were seen in the three months of April-June (22.63% weekday vs weekend 25.71%) whilst similar proportions were seen in the other quarters. The mortality following weekday admission was 0.52% (7,276/1,407,705) compared with 0.77% (986/127,562) following weekend admission. A higher proportion of deaths occurred within 24 h of admission following a weekday admission (1.21% weekday vs 1.01% weekend). The un-adjusted odds ratio for elective patients admitted on the weekend was 1.50 (95% CI 1.40 to 1.60).

In the elective setting, weekend admissions were more likely to be older (mean 57.77 years weekday vs 59.33 years weekend), more likely to be male (49.43% weekday vs 47.12% weekend), more likely to be complex elderly admissions (0.28% weekday vs 0.32% weekend), almost as likely to have a HRG with comorbidity/complication (13.87% weekday vs 13.67% weekend), and were more likely to have a longer median length of stay (1 day weekday vs 3 days weekend), although this difference in length of stay was not reflected in those patients who died (12 days weekday admissions vs 12 days weekend admissions).

### Emergency admissions

Of the 3,105,249 emergency admissions, 76.3% (2,369,316/3,105,249) were admitted on the weekday and 23.7% (735,933/3,105,249) were admitted on the weekend. The mortality following emergency weekday admission was 6.53% (154,761/2,369,316) compared to 7.06% (51,922/735,933) following weekend admission. A higher proportion of deaths occurred within 24 h following a weekend admission (6.53% weekday vs 7.06% weekend). The un-adjusted odds ratio for emergency patients admitted on the weekend is 1.09 (95% CI 1.08 to 1.10).

In the emergency setting, weekend admissions were not more likely to be older (mean 62.21 years weekday vs 61.93 years weekend), more likely to be male (47.44% weekday vs 48.06% weekend), more likely to be complex elderly admissions (7.56% weekday vs 8.15% weekend), almost as likely to have a HRG with comorbidity/complication (35.55% weekday vs 35.17% weekend), and were more likely to have a shorter median length of stay (3 day weekdays vs 4 days weekend), and this was also reflected in the deceased patients (8 days weekday admissions vs 6 days weekend admissions).

### HRG chapter profiles

There were slightly more pronounced differences in HRG Chapter profiles between weekday and weekend admissions in the elective setting than in the emergency setting (see Table 3.)

**Table 3:** HRG profile of weekday and weekend admission (%)

HRG v3.5 Chapter Heading	Elective Admissions (n = 1,535,267)		Emergency Admissions (n = 3,105,249)	
	**Weekday**	**Weekend**	**Weekday**	**Weekend**

N	1,407,705	127,562	2,369,316	735,933

A - Nervous System	34,514 (2.45)	3,584 (2.81)	185,867 (7.84)	60,572 (8.23)

B - Eyes & Periorbita	29,335 (2.08)	1,749 (1.37)	12,159 (0.51)	3,348 (0.45)

C - Mouth, Head, Neck & Ears	107,752 (7.65)	7,032 (5.51)	55,367 (2.34)	20,172 (2.74)

D - Respiratory System	47,411 (3.37)	5,947 (4.66)	333,444 (14.07)	109,629 (14.90)

E - Cardiac Surgery & Primary Cardiac Conditions	95,908 (6.81)	8,884 (6.96)	402,661 (16.99)	121,482 (16.51)

F - Digestive System	168,351 (11.96)	14,358 (11.26)	366,310 (15.46)	105,541 (14.34)

G - Hepato - biliary & Pancreatic System	62,228 (4.42)	4,405 (3.45)	79,026 (3.34)	23,145 (3.14)

H - Musculoskeletal System	270,857 (19.24)	29,756 (23.33)	240,373 (10.15)	86,915 (11.81)

J - Skin, Breast & Burns	89,432 (6.35)	5,862 (4.60)	101,928 (4.30)	28,904 (3.93)

K - Endocrine & Metabolic System	17,449 (1.24)	933 (0.73)	49,752 (2.10)	13,105 (1.78)

L - Urinary Tract & Male Reproductive System	164,496 (11.63)	15,637 (12.26)	187,675 (7.92)	59,143 (8.04)

M - Female Reproductive System	126,808 (9.01)	10,410 (8.16)	57,273 (2.42)	17,029 (2.31)

P - Diseases of Childhood	3,621 (0.26)	350 (0.27)	25,466 (1.07)	8,813 (1.20)

Q - Vascular System	57,639 (4.09)	5,686 (4.46)	30,821 (1.30)	6,707 (0.91)

R - Spinal Surgery & Primary Spinal Conditions	34,468 (2.45)	5,069 (3.97)	37,397 (1.58)	10,492 (1.43)

S - Haematology, Infectious Diseases, Poisoning & Non-Specific Groupings	95,482 (6.78)	7,746 (6.07)	194,325 (8.20)	58,256 (7.92)

T - Mental Health	1,954 (0.41)	154 (0.12)	9,472 (0.40)	2,680 (0.36)

Elective weekend and weekday admissions were, in general comparable, but we saw some differences in a few HRGs. For example a higher percentage of weekend admissions had Chapter H HRGs (19.24% weekday vs 23.33% weekend) but a lower percentage had Chapter C (7.65% weekday vs 5.51% weekend) and J (6.35% weekday vs 4.60% weekend) HRGs. Similarly emergency weekend and weekday admissions were, in general comparable, although we found that a higher percentage of weekend admissions had Chapter H HRGs (10.15% weekday vs 11.81% weekend).

### Statistical modelling

We constructed logistic regression models predicting death in hospital comparing weekend versus weekday admissions for elective and emergency admissions after adjusting for a number of patient case-mix factors and other covariates with reasonable model calibration and discrimination statistics for the 10% sample models (Table 4).

**Table 4:** Model fit statistics for 10% sample and all cases.

Model	Elective Admissions	Emergency Admissions
**10% Sample**		

N	153,036	310,525

Hosmer-Lemeshow Goodness of fit test†	*χ*^2 ^= 2.19; p-value = 0.97	*χ*^2 ^= 4.36; p-value = 0.80

Discrimination (c-statistic)	0.792	0.786

**All cases**		

N	1,535,267	3,105,249

Hosmer-Lemeshow Goodness of fit test†	*χ*^2 ^= 7.37; p-value = 0.50	*χ*^2 ^= 31.78; p-value = 0.0001

Discrimination (c-statistic)	0.786	0.787

†Eight degress of freedom

The models are shown in Table 5 with the last row showing the coefficient for weekend admission. We found that even after case-mix adjustment there was a weekend effect, which was larger in the elective setting (Odds Ratio: 1.32) compared to the emergency setting (Odds Ratio: 1.09). Interestingly, when comparing model coefficients for the covariates between models, we found evidence that the effect of a given covariate differed with setting. For example the odds ratio for complex elderly admission in the elective setting was 16.83 compared with 3.05 in the emergency setting, suggesting that the relationships between these covariates and mortality differs sustainably between elective and emergency settings.

**Table 5:** Statistical modelling results, showing the odds ratio for each variable in the model together with 95% confidence intervals (all p-values < 0.001)

	Elective Admissions (n = 153,036)			Emergency Admissions (n = 3,105,249)		
**Covariate**	**Odds Ratio**	**lower CI**	**upper CI**	**Odds Ratio**	**lower CI**	**upper CI**

Male	1.34	1.28	1.40	1.16	1.13	1.20

Age Category 1: 40-44 years^**†**^	1.34	1.03	1.74	2.99	2.27	3.94

Age Category 2: 45-49 years	2.22	1.78	2.77	4.98	3.91	6.33

Age Category 3: 50-54 years	2.73	2.22	3.35	6.36	5.05	8.02

Age Category 4: 55-59 years	3.94	3.27	4.75	10.25	8.30	12.65

Age Category 5: 60-64 years	5.13	4.31	6.09	13.54	11.08	16.54

Age Category 6: 65-69 years	6.16	5.19	7.30	17.54	14.41	21.34

Age Category 7: 70-74 years	8.71	7.38	10.29	32.03	26.52	38.68

Age Category 8 75-79 years	12.85	10.91	15.13	40.43	33.58	48.68

Age Category 9: 80-84 years	15.92	13.48	18.80	48.95	40.69	58.88

Age Category 10: 85-89 years	21.91	18.44	26.04	58.35	48.51	70.20

Age Category 11: 90-120 years	28.40	23.10	34.91	76.88	63.76	92.70

HRG withcc (vs HRG without cc)	8.20	5.98	11.22	5.39	4.03	7.23

Age Category 1 and HRG withcc	0.88	0.53	1.46	0.79	0.51	1.22

Age Category 2 and HRG withcc	0.74	0.48	1.15	0.47	0.32	0.71

Age Category 3 and HRG withcc	0.46	0.30	0.69	0.56	0.39	0.81

Age Category 4 and HRG withcc	0.49	0.34	0.71	0.43	0.30	0.60

Age Category 5 and HRG withcc	0.40	0.28	0.56	0.41	0.30	0.57

Age Category 6 and HRG withcc	0.35	0.25	0.49	0.36	0.27	0.50

Age Category 7 and HRG withcc	0.16	0.11	0.22	0.10	0.08	0.14

Age Category 8 and HRG withcc	0.12	0.09	0.17	0.11	0.08	0.15

Age Category 9 and HRG withcc	0.13	0.09	0.19	0.12	0.09	0.16

Age Category 10 and HRG withcc	0.14	0.10	0.20	0.13	0.10	0.18

Age Category 11 and HRG withcc	0.17	0.11	0.24	0.14	0.10	0.19

Admission Quarter (Jul-Sep)*	1.09	1.02	1.16	0.96	0.92	1.01

Admission Quarter (Oct-Dec)	1.06	0.99	1.13	0.93	0.89	0.97

Admission Quarter (Jan-Mar)	1.14	1.07	1.22	1.06	1.02	1.11

Complex Elderly	16.83	15.38	18.41	3.05	2.91	3.18

Zero day stay^!^	0.09	0.07	0.11	-	-	-

Weekend vs. Weekday	1.32	1.23	1.41	1.09	1.05	1.13

† Reference category is age 16-39 years. * Reference category is Apr-Jun. Covariates terms with "and" indicate interaction terms. ! Not applicable for emergency admissions

## Discussion

Using about five million hospital discharges from all acute NHS hospitals in England for a year (08/09), we found that after adjustment for a range of risk factors, admission on the weekend was associated with an increased risk of death, which was more pronounced in the elective setting. In the emergency setting the odds increased by 9% (Odds Ratio: 1.09) and in the elective setting the odds increased by 32% (Odds Ratio: 1.32).

Several large database studies have found an increased risk of death for weekend admissions. Aylin et al's [19] study of all emergency admissions to English NHS acute hospitals for the year 2005/2006 (n = 4.3 million emergency admissions), reported a 10% increase in the odds of death following weekend admissions versus weekday admission after statistical adjustment for known confounders [18]. This effect size is consistent with our emergency admissions model despite differences in the case-mix adjustment scheme, whereby they used the Clinical Classification System (CCS) [20] and we adopted HRG codes, although they both ultimate rely on the same HES database. Bell and Redelmeier [21] in a large study (3.8 million emergency admissions in Ontario, Canada) noted that weekend emergency admissions were associated with significantly higher mortality rates for 23 of the 100 leading causes of death and were not associated with significantly lower mortality rates for any of these conditions. A more recent large database study to examine the weekend effect is that of Freemantle et al. [13], who also considered elective and emergency admissions, but not as separate analyses. They used time to event survival models and, unlike other studies in the field, also investigated the risks to patients of being exposed to weekend care irrespective of the day of admission. Freemantle et al. [13] found an increased risk of death for weekend admissions (in elective and emergency settings, Hazard Ratio 1.14 to 1.16 for weekend admissions), but paradoxically they also found that being in hospital over the weekend, irrespective of day of admission, was associated with a reduced risk of death, suggesting that the mechanisms underlying the increased risk for weekend admissions are likely to be complex. Freemantle et al. [13] also incorporated elective admissions into their study but did not undertake separate analyses for elective and emergency admissions and so their findings are not specific to these settings. Our finding that the weekend effect is more pronounced in the elective setting as opposed to the emergency setting appears to be novel.

Numerous smaller studies have investigated the effect of acute weekend admission on hospital mortality [2]. For specific acute conditions or settings [3-20]. A recent review of these studies concluded that the evidence for a weekend effect was mixed - some studies found an effect whilst others did not^2^. Where studies have found an increased risk of death associated with weekend admission, two major explanations have been forwarded [2,4], which are not mutually exclusive: that patients admitted over the weekend are sicker than their weekday counterparts, and/or that patients admitted over the weekend experience poorer quality of care.

Evidence to support the "sicker patients at weekends" hypothesis stems from the non-uniform daily incidence of acute conditions (eg acute myocardial infarction, stroke) reported in some studies [8,10] but not in others [6]. Where weekend and weekday incidence patterns differ this can lead to selection bias [2]. One possible mechanism for differential incidence is that patients with less severe acute illness may avoid a weekend admission, choosing instead to present on a weekday. This would mean that weekend patients are sicker but this mechanism can potentially operate both ways - patients with less severe illness may choose to present on a weekday as a way of avoiding a weekend admission. We did find some evidence of differential levels of complex elderly admissions between weekend and weekday admissions in the elective and emergency setting, although we expect that the statistical model will adequately control for such differences providing the underlying relationship between patient sickness and mortality is the same for weekend and weekday admission [22]. Whilst Becker has called for further condition specific studies as a way to minimise selection bias in the study of the weekend effect [2], our study along with others [2-18] suggest that there is generic dimension to the weekend effect, which according to our findings, is more marked in the elective setting. Furthermore, since the sicker patient's hypothesis relies on non-uniform daily incidence of acute conditions, this explanation is less applicable to the elective setting where admission is planned.

Direct evidence that emergency admissions admitted on the weekend may experience suboptimal care has been reported in some studies (examining specific acute conditions such acute myocardial infarction, stroke) but not others [6,8]. Where suboptimal care has been found it was mostly due to delay in treatment [2]. However the extent and nature of possible suboptimal care in the elective weekend versus weekday setting remains somewhat under explored, perhaps because previous work has focused primarily on emergency admissions.

Our study, like several other studies [3-20] is void of any direct measurement of care and it would be premature to presume that apparently adequate case-mix adjustment safely rules out the "sicker weekend patients" hypothesis and rules in the "quality of care" hypothesis [23]. For instance, it is worth noting that if patients admitted over the weekend have less comprehensive clinical coding (perhaps because case-notes are less complete for weekend admissions), then such differential measurement error could potentially undermine the case-mix adjustment in our model, because the "sickness" of weekend admissions may have been systematically underestimated. Or, for emergency admissions, if provision for palliative care in the community is reduced over the weekend then hospitals will see an increased mortality over the weekend not because of poorer quality of care but because the mortality burden is displaced at the weekends. Indeed it is also worth noting that case-mix adjustment itself is not without risks of bias [21] and as recommended by Nicholls [21] we systematically checked for interaction effects between our primary covariate (weekend admission) and the other covariates in the model. Nevertheless, further studies to determine the relationship between the processes of care for weekend versus weekday admissions especially in the elective setting appear justifiable even though we know from a systematic review that the links between excess mortality and quality of care are not always reliable [24].

There are some issues with our approach to modelling which merit discussion. We did not combine elective and emergency admissions into the same model as Freemantle et al. [13] have done. Our rationale was that elective and emergency admissions are generated by different processes and the material differences in coefficients for the same covariate would appear to support. Furthermore, as recommended by Jones [15] and noted in another study where emergency admissions were analysed, [25] we excluded zero stay emergency admission that were discharged alive, from our data but note that other studies using NHS data [13-18] did not make special allowances for these zero stay admissions. During model development we found that model stability and healthy goodness-of-fit statistics were achieved after including an interaction between "withcc" (with comorbidities and/or complications), but this was less surprising because HRG codes which include a "with cc" description may also include an age criterion (e.g. aged < 69 years). We did not exclude bank holidays from the analysis, because there are only eight bank holidays in England and exclusion (or indeed special attention e.g. as a covariate) was not likely to seriously impact on our findings. We developed an adequately fitting model using an arbitrary 10% random sample, but our overall model calibration statistics were statistically significant for the emergency admissions (but not the elective admissions), presumably because of the very large sample sizes involved. Our approach to case-mix adjustment did not incorporate any specific disease groups because this was not our primary hypothesis. This does not mean that disease specific models are not warranted. Nevertheless we emphasise that since desktop based exercises, such as ours, are generally void of any direct measures of care their ability to inform efforts to improve care over the weekends is limited [2,4]. Since the formulation of interventions to address deficiencies in care over the weekends requires painstaking detective work to determine the causal relationships between processes of care on the weekend and mortality, we would therefore prioritise this type of challenging research activity, commencing with the elective setting.

## Conclusions

Weekend admission appears to be an independent risk factor for dying in hospital and this risk is more pronounced in the elective setting. Given the planned nature of elective admissions, as opposed to the unplanned nature of emergency admissions, it would seem less likely that this increased risk in the elective setting is attributable to unobserved patient risk factors. Further work to understand the relationship between weekend processes of care and mortality, especially in the elective setting, is required.

## Competing interests

The authors declare that they have no competing interests.

## Authors' contributions

SK conceived of the study and undertook preliminary analysis. MAM and GR developed the work and undertook further statistical analyses. AS also developed the work and helped with writing and interpretation. All authors contributed to the manuscript and have all read and approved the final manuscript.

## Appendix

To mitigate against spurious p-values resulting from the very large sample sizes involved in our study, we undertook model development using a 10% stratified random sample from the elective and emergency admissions. Modelling results from the 10% random samples are shown in Table 6.

**Table 6:** Statistical modelling results, showing the odds ratio for each variable in the model together with 95% confidence intervals (all p-values < 0.001)

	Elective Admissions			Emergency Admissions		
**Covariate**	**OR**	**lower CI**	**upper CI**	**OR**	**lower CI**	**upper CI**

Male	1.31	1.14	1.51	1.16	1.13	1.20

Age Category 1: 40-44 years^††^	0.73	0.31	1.67	2.99	2.27	3.94

Age Category 2: 45-49 years	0.86	0.40	1.83	4.98	3.91	6.33

Age Category 3: 50-54 years	1.33	0.70	2.50	6.36	5.05	8.02

Age Category 4: 55-59 years	2.86	1.74	4.72	10.25	8.30	12.65

Age Category 5: 60-64 years	2.63	1.62	4.26	13.54	11.08	16.54

Age Category 6: 65-69 years	4.46	2.85	6.98	17.54	14.41	21.34

Age Category 7: 70-74 years	4.82	3.08	7.54	32.03	26.52	38.68

Age Category 8 75-79 years	7.92	5.15	12.20	40.43	33.58	48.68

Age Category 9: 80-84 years	10.22	6.59	15.87	48.95	40.69	58.88

Age Category 10: 85-89 years	13.43	8.43	21.40	58.35	48.51	70.20

Age Category 11: 90-120 years	21.60	12.38	37.70	76.88	63.76	92.70

HRG withcc (vs. HRG without cc)	3.03	0.91	10.03	5.39	4.03	7.23

Age Category 1 and HRG withcc	1.00			0.79	0.51	1.22

Age Category 2 and HRG withcc	2.75	0.54	13.95	0.47	0.32	0.71

Age Category 3 and HRG withcc	2.87	0.69	11.86	0.56	0.39	0.81

Age Category 4 and HRG withcc	0.87	0.22	3.46	0.43	0.30	0.60

Age Category 5 and HRG withcc	1.24	0.34	4.57	0.41	0.30	0.57

Age Category 6 and HRG withcc	0.79	0.22	2.84	0.36	0.27	0.50

Age Category 7 and HRG withcc	0.47	0.13	1.67	0.10	0.08	0.14

Age Category 8 and HRG withcc	0.33	0.09	1.15	0.11	0.08	0.15

Age Category 9 and HRG withcc	0.31	0.09	1.11	0.12	0.09	0.16

Age Category 10 and HRG withcc	0.54	0.15	1.91	0.13	0.10	0.18

Age Category 11 and HRG withcc	0.48	0.12	1.89	0.14	0.10	0.19

Admission Quarter (Jul-Sep)^†^	1.12	0.91	1.36	0.96	0.92	1.01

Admission Quarter (Oct-Dec)	1.00	0.81	1.23	0.93	0.89	0.97

Admission Quarter (Jan-Mar)	1.18	0.97	1.44	1.06	1.02	1.11

Complex Elderly	17.57	13.25	23.30	3.05	2.91	3.18

Zero day stay	0.08	0.04	0.16	-	-	-

Weekend vs. Weekday	1.44	1.16	1.77	1.09	1.05	1.13

† Reference category is age 16-39 years. * Reference category is Apr-Jun. Covariates terms with "and" indicate interaction terms. ! Not applicable for emergency admissions

## Pre-publication history

The pre-publication history for this paper can be accessed here:

http://www.biomedcentral.com/1472-6963/12/87/prepub

## References

[B1] The Audit Commission Medical Staffing (report), Acute hospital portfolio 2002 London: 8 Audit Commission

[B2] Becker D Weekend hospitalisation and mortality: a critical review Expert rev pharmacoecon outcomes res 2008 8 1 23 26 10.1586/14737167.8.1.23 20528352

[B3] Schmulewitz L Proudfoot A Bell D The impact of weekends on outcome for emergency patients Clin Med 2005 5 6 621 625 16411359 10.7861/clinmedicine.5-6-621 PMC4953143

[B4] Halm EA Chassin MR Why do hospital death rates vary? N Engl J Med 2001 9 692 694 11547726 10.1056/NEJM200108303450911

[B5] Cram P Hillis SL Barnett M Rosenthal GE Effects of weekend admission and hospital teaching status on in-hospital mortality Am J Med 2004 117 151 157 10.1016/j.amjmed.2004.02.035 15276592

[B6] Becker DJ Quality of Care and Mortality. Do Hospitals Provide Lower Quality Care on Weekends? Health Res Ed Trust 2007 42 4 1589 1612 10.1111/j.1475-6773.2006.00663.x PMC1955270 17610439

[B7] Saposnik G Baibergenova A Bayer N Hachinski V Weekends: A Dangerous Time for Having a Stroke? Stroke 2007 38 1211 1215 10.1161/01.STR.0000259622.78616.ea 17347472

[B8] Kostis W Demissie K Marcella S Yu H Wilson A Moreyra A Weekend versus weekday admission and mortality from myocardial infarction N Engl J Med 2007 356 1099 1109 10.1056/NEJMoa063355 17360988

[B9] Barnett M Kaboli P Sirio C Rosenthal G Day of the week of intensive care admission and patient outcomes Med Care 2002 40 6 530 539 10.1097/00005650-200206000-00010 12021679

[B10] Saposnik G Bailbergenova A Bayer N Hachinksi V Weekends: a dangerous time for having a stroke Stroke 2007 38 1211 1215 10.1161/01.STR.0000259622.78616.ea 17347472

[B11] Marco J Barba R Plaza S Losa JE Canora J Zapatero A Analysis of the mortality of patients admitted to internal medicine wards over the weekend Am J Med Qual 2010 25 4 312 318 10.1177/1062860610366031 20484660

[B12] Barba R Zapatero A Emilio Losa J Marco J Plaza S Rosado C Canora J The impact of weekends on outcome for Acute exacerbations of COPD Eur Respir J 2012 39 1 46 50 10.1183/09031936.00013211 21659418

[B13] Freemantle N Weekend hospitalization and additional risk of death. An analysis of inpatient data J R Soc Med 2012 1 11 doi:10.1258/jrsm.2012.120009 10.1258/jrsm.2012.120009 PMC3284293 22307037

[B14] Hospital Episode Statistics (HES) online http://www.hesonline.nhs.uk/

[B15] Jones R Trends in emergency admissions Br J Healthc Manag 2009 15 4 188 196

[B16] Benton P Evans H Light S Mountney M Sanderson H Anthony P The development of Healthcare Resource Groups - version 3 J Public Health Med 1998 20 3 351 358 9793902 10.1093/oxfordjournals.pubmed.a024779

[B17] Fetter R Shin Y Freeman J Averill R Thompson D Case Mix Definition by Diagnosis-Related Groups Medical Care 1980 18 2 supplement 7188781

[B18] StataCorp, StataCorp LP Stata Statistical Software 2011 College Station, TX: Release 12

[B19] Aylin P Yunus A Bottle A Weekend mortality for emergency admissions. A large, multicenter study Qual Saf Health Care 2010 19 213 217 10.1136/qshc.2008.028639 20110288

[B20] Clinical Classifications Software for ICD-10 Data: 2003 Software and User's Guide 2003 Agency for Healthcare Research and Quality, Rockville, MD http://www.ahrq.gov/data/hcup/icd10usrgd.htm

[B21] Bell CM Redelmeier DA Mortality among patients admitted to hospital on weekends as compared with weekdays NEJM 2001 345 9 663 668 10.1056/NEJMsa003376 11547721

[B22] Nicholl J Case-mix adjustment in non-randomised observational evaluations: the constant risk fallacy J Epidemiol Comm Health 2007 61 1010 1013 10.1136/jech.2007.061747 PMC2465605 17933961

[B23] Lilford R Mohammed MA Spiegelhalter D Thomson R Use and misuse of process and outcome data in managing performance of acute medical care: avoiding institutional stigma Lancet 2004 363 1147 54 10.1016/S0140-6736(04)15901-1 15064036

[B24] Pitches D Mohammed MA Lilford R What is the empirical evidence that hospitals with higher-risk adjusted mortality rates provide poorer quality of care? A systematic review of the literature BioMed Centr Health Serv Res 2007 7 91 http://www.biomedcentral.com/1472-6963/7/91. 10.1186/1472-6963-7-91 PMC1924858 17584919

[B25] Mohammed MA Deeks J Evidence of methodological bias in hospital standardized mortality ratios: retrospective database study of English hospitals BMJ 2009 338 b780 10.1136/bmj.b780 19297447 PMC2659855

